# Aberrantly glycosylated IgG elicits pathogenic signaling in podocytes and signifies lupus nephritis

**DOI:** 10.1172/jci.insight.147789

**Published:** 2021-05-10

**Authors:** Rhea Bhargava, Sylvain Lehoux, Kayaho Maeda, Maria G. Tsokos, Suzanne Krishfield, Lena Ellezian, Martin Pollak, Isaac E. Stillman, Richard D. Cummings, George C. Tsokos

**Affiliations:** 1 Department of Medicine and; 2 Department of Surgery, Beth Israel Deaconess Medical Center and Harvard Medical School, Boston, Massachusetts, USA.; 3 Beth Israel Deaconess Medical Center Glycomics Core, Boston, Massachusetts, USA.; 4 Department of Pathology, Beth Israel Deaconess Medical Center, Boston, Massachusetts, USA.

**Keywords:** Autoimmunity, Immunology, Calcium signaling, Glycobiology, Immunoglobulins

## Abstract

Lupus nephritis (LN) is a serious complication occurring in 50% of patients with systemic lupus erythematosus (SLE) for which there is a lack of biomarkers, a lack of specific medications, and a lack of a clear understanding of its pathogenesis. The expression of calcium/calmodulin kinase IV (CaMK4) is increased in podocytes of patients with LN and lupus-prone mice, and its podocyte-targeted inhibition averts the development of nephritis in mice. Nephrin is a key podocyte molecule essential for the maintenance of the glomerular slit diaphragm. Here, we show that the presence of fucose on N-glycans of IgG induces, whereas the presence of galactose ameliorates, podocyte injury through CaMK4 expression. Mechanistically, CaMK4 phosphorylates NF-κB, upregulates the transcriptional repressor SNAIL, and limits the expression of nephrin. In addition, we demonstrate that increased expression of CaMK4 in biopsy specimens and in urine podocytes from people with LN is linked to active kidney disease. Our data shed light on the role of IgG glycosylation in the development of podocyte injury and propose the development of “liquid kidney biopsy” approaches to diagnose LN.

## Graphical abstract

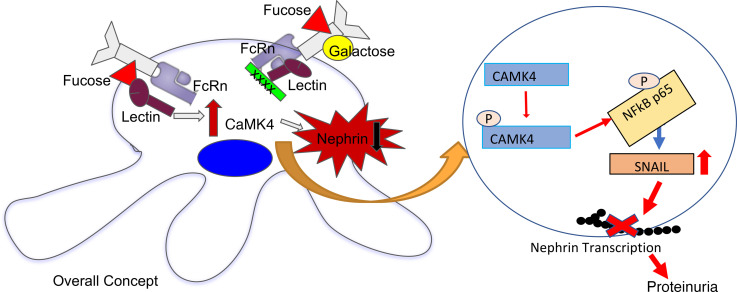

## Introduction

Lupus nephritis (LN) represents one of the most serious complications of systemic lupus erythematosus (SLE) and is associated with significant morbidity and mortality. People with LN have a higher standardized mortality ratio compared with those without LN (6–6.8 versus 2.4–3, respectively) (1–4), and up to 30% of them develop end-stage renal disease (5), which is also a predictor of poor outcome (6–10). Achievement and maintenance of remission of LN improves the 10-year survival from 46% to 95% (11).

The pathogenesis of LN involves the deposition of circulating or in situ–formed immune complexes in different areas of the glomerulus, leading to activation of components of the innate and adaptive immune system (12–16). However, the exact immunopathogenesis of LN is still elusive, and the inciting events that lead to resident kidney cell injury and organ failure are largely unknown. It has been proposed that podocyte injury occurs early in LN after deposition of immune complexes and precedes irreversible glomerular damage (17–19). Depletion of more than 30% of podocytes causes glomerular destabilization, which leads to eventual glomerulosclerosis and has been associated with the severity of LN (20, 21).

Calcium signaling plays a key role in the maintenance of the actin cytoskeleton of cells (22–28). We previously demonstrated that calcium/calmodulin kinase IV (CaMK4) expression is increased in podocytes of lupus prone MRL*/lpr* mice and that exposure of cultured human podocytes to IgG from individuals with SLE leads to CaMK4 upregulation (19, 29). CaMK4 is known to shuttle between the cytoplasm and the nucleus. It has been previously shown that CaMK4 is phosphorylated in the cytoplasm and can then use importin α to shuttle to the nucleus (30–32). CaMK4 requires phosphorylation on a threonine residue located at the activation loop. This is generated by the upstream Ca^2+^/CaM-dependent kinase kinases located mainly in the cytoplasm (30, 31). We have previously demonstrated that CaMK4 is localized mainly in the nuclei of spleen cells from MRL/*lpr* mice compared with the spleen cells of the Fas-intact control mice MRL/MPJ in which virtually all CaMK4 is localized in the cytoplasm (33). Genetic or pharmacologic inhibition of CaMK4 prevents development of nephritis in lupus-prone mice (34, 35). Interestingly, inhibition of CaMK4 — in podocytes only — prevents immune complex deposition and preserves renal function, despite systemic autoimmunity remaining intact (19).

Nephrin is a key podocyte molecule that is essential for the maintenance of normal slit diaphragm structure (36). Loss of nephrin during development leads to congenital nephrotic syndrome in children (37), and reduction of nephrin expression is often observed in adult kidney diseases, including proliferative LN, diabetic nephropathy, and HIV-associated nephropathy (38–44). The critical role of nephrin in podocyte function has been confirmed in different animal models where knockdown of nephrin aggravates the progression of kidney disease (38–46). More importantly, podocyte-specific genetic variants, such as a homozygous variant of *NPHS1*, which encodes nephrin, have been described in patients with treatment-resistant LN (47).

The significant morbidity and mortality associated with LN highlights the importance of identifying patients with SLE who are likely to develop kidney disease and can benefit from early therapeutic intervention. Here, we show that overexpression of CaMK4 in renal biopsy material represents a biomarker of active LN. More importantly, IgG from individuals with LN, but not from those with SLE without clinical evidence of LN, upregulates CaMK4 expression in cultured podocytes. In parallel, podocytes present in the urine of individuals with active LN display increased CaMK4 levels. Mechanistically, we demonstrate that the presence of fucose on N-linked glycans in undergalactosylated IgG are responsible for the increased expression of CaMK4 in podocytes. CaMK4 represses nephrin transcription through a signaling pathway that involves CaMK4-induced phosphorylation of NF-κB and upregulation of SNAIL, the transcriptional repressor of nephrin. Our studies suggest approaches and tools that could limit the need for kidney biopsies in diagnosing LN, as well as the consideration of IgG glycosylation modulators to prevent or reverse the development of LN.

## Results

### IgG from SLE patients with LN, but not without LN, increases CaMK4 expression in podocytes.

**Figure 1:**
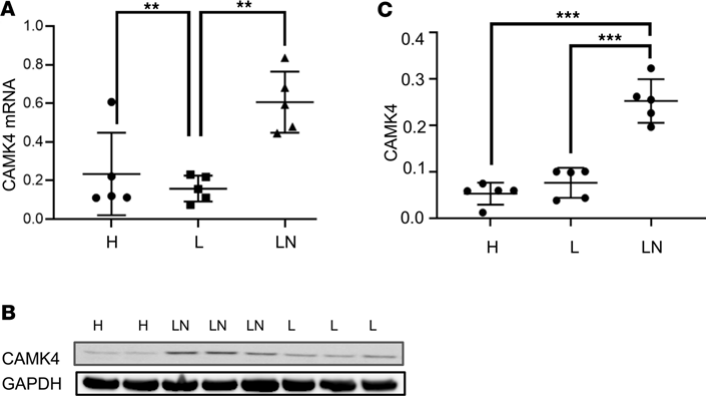
IgG from patients with LN upregulates CaMK4 in podocytes. Podocytes in culture were exposed to IgG from individuals with lupus (L) with no kidney involvement, active lupus nephritis (LN), or healthy controls (H). (**A** and **B**) CaMK4 mRNA (CaMK4/GAPDH) after RNA extraction (**A**) and CaMK4 protein expression (representative experiment) (**B**) from cell lysates was measured. (**C**) Densitometric quantification of Western blot (CaMK4/GAPDH) data from 3 independent experiments was performed (*n* = 5 individuals per group, total *n* = 15).

Previously, we demonstrated that IgG from patients with SLE, but not from healthy subjects, causes upregulation of CaMK4 in podocytes followed by podocyte injury (16, 19). Because not all patients with SLE develop LN (9), we sought to evaluate whether IgG from SLE patients without evidence of LN can upregulate CaMK4 in a similar manner. To this end, we exposed cultured human podocytes to IgG from healthy subjects or SLE patients with and without LN. Fifteen samples (5 per group) from age- and sex-matched individuals were used (Table 1). We found that CaMK4 expression at the protein and mRNA levels increased only in podocytes cultured in the presence of IgG from patients with active LN and not in podocytes exposed to IgG from healthy controls or SLE patients without LN (Figure 1, A–C). In parallel, we noted that the expression of the IL-17 receptor and IL-23 was increased in podocytes exposed to IgG from patients with LN but not from SLE patients without clinical nephritis and healthy controls. The expression of the costimulatory molecule CD86 but not of CD80 followed the same pattern, whereas the expression of TNF-α and IL-6 was not affected significantly (Supplemental Figure 1; supplemental material available online with this article; https://doi.org/10.1172/jci.insight.147789DS1). These data demonstrate that IgG from SLE patients with LN, but not from those without LN, induce the expression of genes that have been linked to the pathogenesis of LN (48–57). IgG from healthy volunteers did not upregulate CaMK4 expression (Supplemental Figure 2).

### Fucose enhances, whereas galactose decreases, the ability of IgG to upregulate CaMK4.

**Figure 2:**
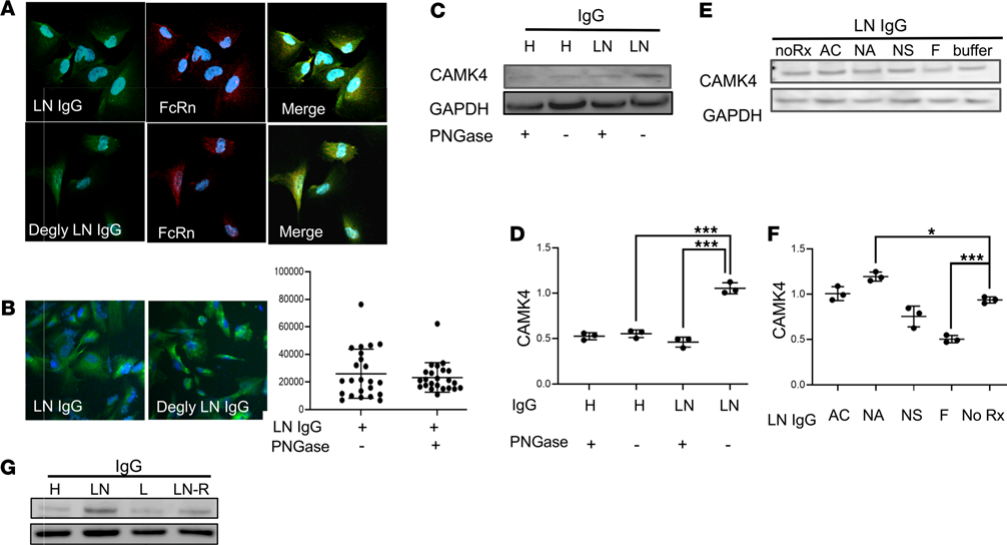
Treatment of IgG with α-fucosidase prevents the upregulation of CaMK4 in podocytes. (**A**) Both deglycosylated (Degly) and untreated IgG from individuals with LN colocalize with neonatal Fc receptor (FcRn) in human podocytes (original magnification, 200×). (**B**) Intensity of Alexa Fluor–tagged PNGase F–treated (deglycosylated) and untreated LN-derived IgG in podocytes was analyzed by immunofluorescence staining (20×). (**C**) CaMK4 expression was evaluated after exposure of podocytes to deglycosylated or untreated IgG from healthy controls (H) or patients with LN (a representative experiment is shown). (**D**) Densitometry was performed for quantification of the results in **C** (3 independent experiments were performed). Data are shown as mean ± SEM. ***P < 0.01, by 1-way ANOVA with Bonferroni post hoc test correction. (**E**) CaMK4 expression in podocytes after exposure to IgG derived from a patient with LN before (noRx) or after treatment with β-*N*-acetylglucosaminidase (AC), Neuraminidase A (NA), Neuraminidase S (NS), or α-fucosidase (F). (**F**) Densitometry was performed for quantification of the results in **E**. Data are shown as mean ± SEM. *P < 0.05; ***P < 0.01, by 1-way ANOVA with Bonferroni post hoc test correction (3 independent experiments utilizing IgG from 3 different individuals were performed for each representative experiment displayed above). (**G**) CaMK4 expression was evaluated after exposure of podocytes to IgG differing in glycosylation profiles from individuals with LN in remission, those with SLE with and without nephritis, and those without disease.

Because N-glycans regulate effector functions of IgG (58, 59), we hypothesized that modifications in IgG glycosylation are responsible for its ability to upregulate CaMK4 in podocytes. Accordingly, we used PNGase F to remove enzymatically N-linked glycans from IgG from healthy individuals and patients with active LN and evaluated its ability to increase CaMK4 expression in cultured podocytes. In a previous study, we had demonstrated that IgG from individuals with SLE upregulates CaMK4 after it enters podocytes using the neonatal Fc receptor (FcRn) (29). Therefore, we first established that deglycosylated IgG binds to FcRn (Figure 2A) and enters podocytes at amounts equivalent to those of nonmodified IgG (Figure 2B). In contrast to the nonmodified IgG, deglycosylated IgG from patients with LN did not cause upregulation of CaMK4 in podocytes (Figure 2, C and D), suggesting that upregulation of CaMK4 requires the presence of N-glycans on IgG. We also observed that podocytes exposed to nonmodified IgG from patients with LN, but not to deglycosylated LN IgG, were hypermotile as assessed in transwell migration assays (Supplemental Figure 3). Furthermore, actin fibers were fragmented and condensed in podocytes exposed to IgG from patients with LN but not after exposure to deglycosylated IgG (Supplemental Figure 3). Nephrin expression was reduced in podocytes after exposure to IgG from patients with LN compared with podocytes exposed to deglycosylated IgG from patients with LN and IgG from healthy volunteers (Supplemental Figure 3C). Of note, we have previously demonstrated that CaMK4 modulates the actin cytoskeleton and motility in podocytes (19, 60).

To dissect the role of distinct glycan residues in upregulating CaMK4 and in podocyte injury, we treated IgG from patients with LN with specific glycosidases. We found that removal of fucose, following treatment with α-fucosidase, diminished the ability of LN-derived IgG to upregulate CaMK4 (Figure 2, E and F). Next, we treated IgG with Endo-S, an IgG-specific endoglycosidase that acts on the chitobiose core, leaving the core GlcNAc-Asn unit intact and, hence, the core fucose on IgG preserved as Fuc-GlcNAc-Asn (Supplemental Figure 4B). We found that LN-derived IgG treated with Endo-S, maintained its ability to increase CaMK4 in podocytes, but additional treatment with fucosidase abrogated this ability (Supplemental Figure 4A and Supplemental Figure 3B). To further explore whether Fc fragment containing bound fucose influenced CaMK4 expression, we exposed podocytes to IgG that had been modified artificially to be heavily fucosylated or afucosylated at the Fc segment. Indeed, only fucosylated IgG was able to upregulate CaMK4 (Figure 2G and Supplemental Figure 4C). The removal of fucose from IgG was confirmed by lectin blot analysis using Aleuria aurantia lectin (AAL), which binds specifically to fucose (61) (Supplemental Figure 4D).

**Figure 3:**
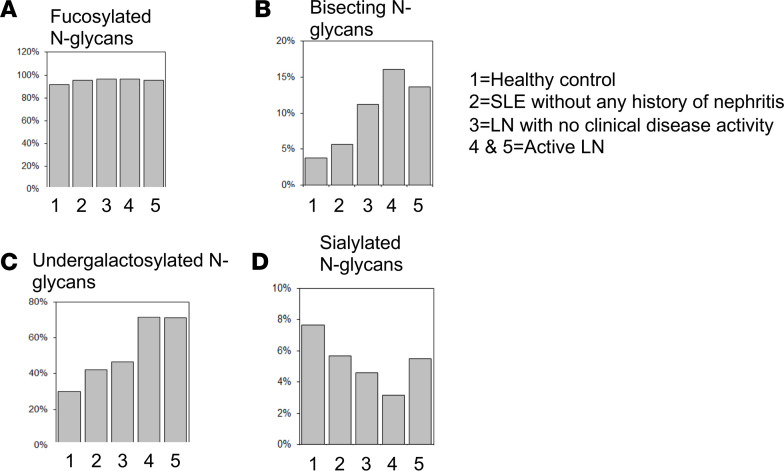
The IgG N-glycome in individuals with active LN differs from healthy controls and SLE patients without kidney disease. Mass spectrometric quantification analysis of IgG from healthy controls (1), SLE patient with no kidney involvement (2), SLE patient with LN in remission (3), and SLE patients with active LN (4 and 5). Fucosylated N glycans (**A**), bissected N-glycans (**B**), undergalactosylated N glycans (**C**), and sialylated N glycans (**D**) (*n* = 5).

To obtain additional details on N-glycan changes in IgG from individuals with LN, we analyzed and compared the IgG N-glycome of healthy controls, individuals with SLE with and without nephritis, and those with a history of LN without active clinical disease following treatment, using mass spectrometry. Surprisingly, the relative fucosylation of IgG was similar between healthy controls (97.73%) and SLE patients (96.065%) with and without nephritis (95.36% without nephritis) (Figure 3A). IgG from individuals with nephritis displayed an increase in relative abundance of bisecting N-glycans and nongalactosylated N-glycans compared with healthy individuals and patients with SLE without active LN (Figure 3, B and C). Specifically, we found that 71.35% of IgG from patients with LN lacked galactose, while only 42.11% of IgG in SLE without nephritis and 46.52% of IgG in LN in remission was agalactosylated. Lack of galactose was noted in 30.06% of IgG from healthy controls. There was no significant difference in sialylated glycans among the tested samples (Figure 3D).

**Figure 4:**
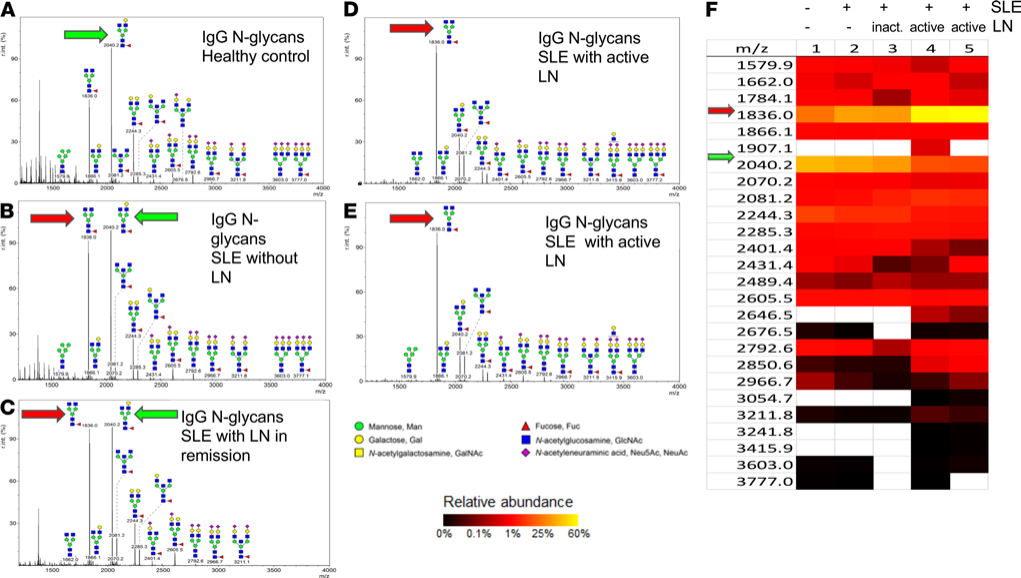
IgG from individuals with active LN is undergalactosylated compared with IgG from healthy controls and individuals with SLE without kidney disease. (**A**–**E**) Representative images of mass spectrometric analysis of IgG from: healthy subject (**A**), SLE patient with no kidney involvement (**B**), SLE patient with LN in remission (**C**), SLE patient with active LN (**D** and **E**). (**F**) Heatmap displaying glycan moieties of different masses for each sample. Red arrow represents the glycan most prevalent in active LN, while green arrow indicates the glycan most prevalent in healthy controls (*n* = 5).

When examining the glycan profiles to identify the most prevalent glycan bound to IgG in patients with LN, we found that a nongalactosylated, core-fucosylated N-glycan (1836.0 *m/z*) was the most abundant N-glycan (~57%) in individuals with active LN (Figure 4, D–F), in contrast to healthy controls in whom a galactosylated, core-fucosylated N-glycan (2040.2 *m/z*) was the most abundant (~42%) (Figure 4, A and F, and Supplemental Figure 5C). Interestingly, individuals with SLE and nephritis in remission or without any kidney involvement showed a relatively moderate abundance (~37%) of both moieties (Figure 4, B, C, and F), whereas patients with active LN had the lowest abundance (~17%) of the galactosylated, core-fucosylated N-glycan (2040.2 *m/z*).

**Figure 5:**
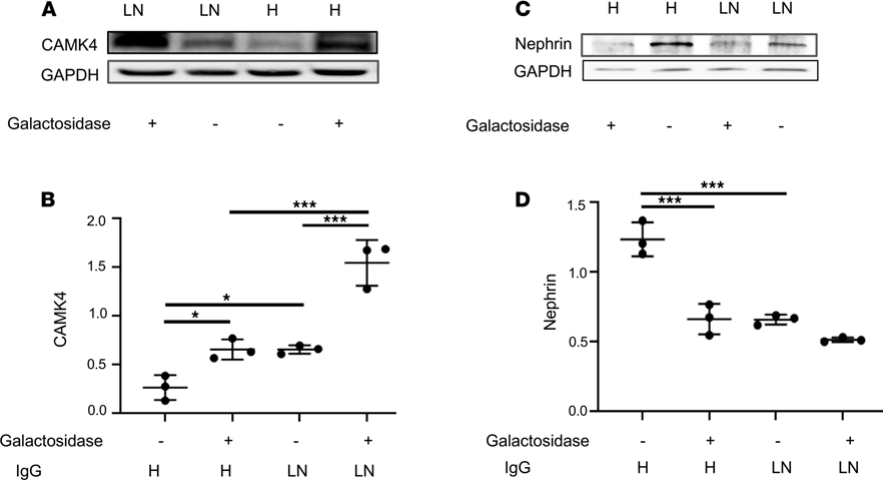
The presence of galactose on IgG has a protective role in podocyte injury. (**A**) CaMK4 expression in podocytes after exposure to untreated IgG and β-galactosidase–treated IgG from healthy controls (H) and individuals with LN. (**B**) Densitometry was performed for quantification of results in **A**. (**C**) Nephrin expression in podocytes after exposure to untreated IgG and β-galactosidase–treated IgG from healthy controls (H) and individuals with LN. (**D**) Densitometry was performed for quantification of the results in **C**. Data are shown as mean ± SEM. *P < 0.05; ***P < 0.01, by 1-way ANOVA with Bonferroni post hoc test correction (3 independent experiments utilizing IgG from 3 different individuals were performed for each representative experiment displayed above).

Because IgG from patients with LN display a characteristically decreased galactosylation pattern, we considered that, while fucose is responsible for podocyte injury, galactose may have a protective effect on the induction of CaMK4 in podocytes. To address this possibility, we studied CaMK4 expression in podocytes exposed to IgG from healthy individuals and patients with active LN before and after treatment with β1,4-galactosidase to remove the galactose residue. We found that after β-galactosidase treatment, IgG from healthy controls gained the ability to upregulate CaMK4 (Figure 5, A and B). Furthermore, IgG from individuals with LN displayed an enhanced ability to cause CaMK4 upregulation after treatment of IgG with β-galactosidase (Figure 5, A and B).

To determine whether β-galactosidase–treated IgG treatment can cause podocyte injury, we analyzed the expression of nephrin, a podocyte slit diaphragm protein important in cell function and viability (62). Decrease in nephrin expression is known to precede podocyte loss and is linked to progression of kidney disease (63). We found that nephrin expression was downregulated in podocytes exposed to β-galactosidase–treated IgG from healthy controls, compared with untreated IgG, and reached levels comparable with those in podocytes exposed to IgG from patients with LN (Figure 5, C and D).

Since removal of galactose from IgG enhanced fucose-induced CaMK4 regulation, we evaluated whether β-galactosidase treatment enhanced accessibility of fucose on IgG. Indeed, using an AAL ELISA, we found that more AAL was bound on IgG after the removal of galactose residues, suggesting enhanced accessibility to the fucose on IgG (Supplemental Figure 5A). Removal of galactose from IgG was confirmed by lectin blot analysis using Erythrina cristagalli lectin (ECL), which has a specificity for terminal β-linked galactose residues (61) (Supplemental Figure 5B).

### CaMK4 regulates nephrin transcription through NF-κB (p65) phosphorylation and enhances SNAIL transcription.

**Figure 6:**
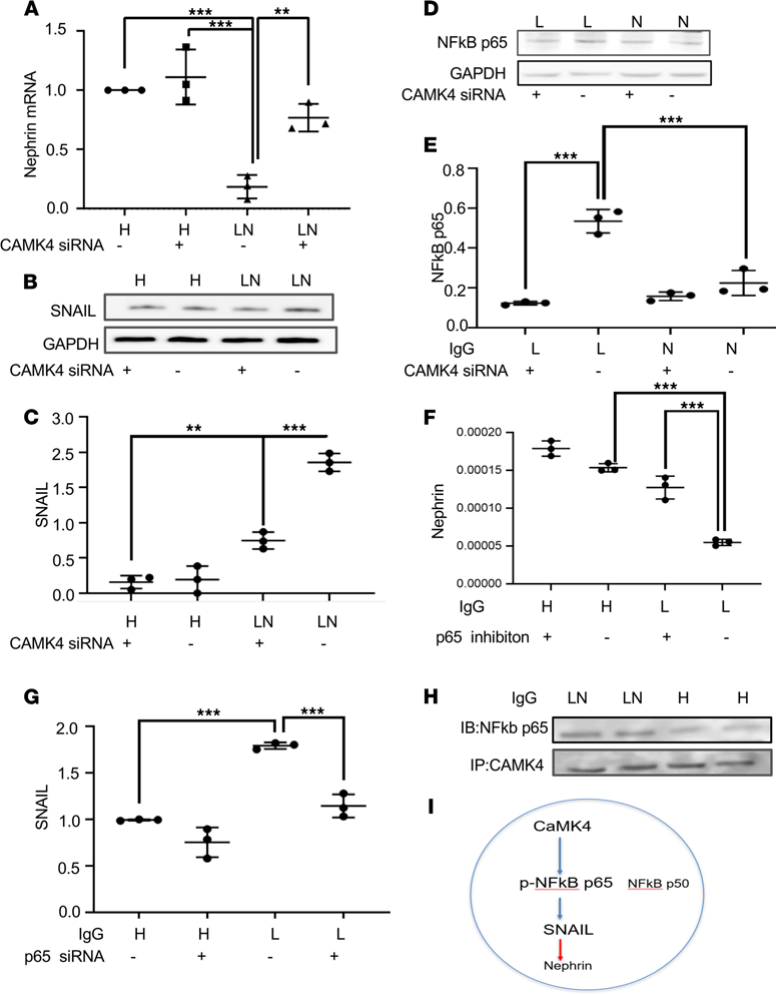
CaMK4 regulates nephrin transcription through NF-κb/p65 activation and SNAIL upregulation. Podocytes were cultured in the presence of IgG from healthy controls or individuals with LN. (**A**) Nephrin mRNA was decreased in podocytes after exposure to IgG from individuals with LN in a CaMK4-dependent manner. (**B**–**E**) Western blot was utilized to measure the expression of SNAIL protein (**B** and **C**) and NF-κB (p65) (**D** and **E**) in the presence or absence of CaMK4 siRNA. (**F** and **G**) Nephrin mRNA (**F**) and SNAIL mRNA (**G**) was quantified in podocytes after exposure to IgG from healthy controls and from patients with LN in the presence or absence of NF-κB (p65). (**H**) CaMK4 was immunoprecipitated from podocytes after exposure to IgG from individuals with LN and healthy controls, and it was immunoblotted (IB) with an antibody against p65. (**I**) Schematic illustration of the proposed mechanism whereby CaMK4 phosphorylates and activates NF-κB (p65), which upregulates SNAIL transcription, leading to repression of nephrin transcription.

Because we have previously shown that CaMK4 suppresses nephrin protein and mRNA expression (19), we conducted a set of experiments to define the involved mechanisms. Using siRNA to silence CaMK4, we found that nephrin transcription was suppressed in podocytes exposed to IgG from patients with LN (Figure 6A). Because SNAIL is a canonical repressor of nephrin transcription (64), we evaluated whether CaMK4 downregulates nephrin transcription through SNAIL. We found that SNAIL protein and mRNA expression increased upon exposure of podocytes to IgG from patients with LN in a CaMK4-dependent manner as its expression failed to increase when CaMK4 was silenced (Figure 6, B and C, and Supplemental Figure 6A). Since SNAIL transcription is regulated by NF-κB (65), which is phosphorylated by CaMK4 (66), we explored a possible interaction of CaMK4 and NF-κB. NF-κB consists of the p65 (RelA) subunit, which contains a transactivation domain responsible for the transcriptional activity, and the p50 subunit, which does not contain a transactivation domain and — in its homodimeric form — acts as a transcriptional repressor (67, 68). We found that the levels of p65 increased in podocytes after exposure to IgG from individuals with LN and decreased in the presence of CaMK4 siRNA (Figure 6, D and E). We also observed that, upon silencing NF-κB (p65), nephrin transcription was preserved (Figure 6F) and SNAIL levels did not increase in podocytes exposed to IgG from patients with LN (Figure 6G). We further immunoprecipitated CaMK4 in podocytes after exposure to normal or LN IgG and blotted the immunoprecipitate with p65 and p50 antibodies. We observed that, while p65 interacted with CaMK4 (Figure 6H), no significant interaction was noted with the p50 subunit (Supplemental Figure 6B). Our experiments reveal a pathway whereby IgG from LN upregulates CaMK4, which suppresses nephrin transcription through activation of NF-κB and upregulation of the nephrin transcriptional repressor SNAIL (Figure 6I).

### CaMK4 expression in renal biopsies identifies active LN.

**Figure 7:**
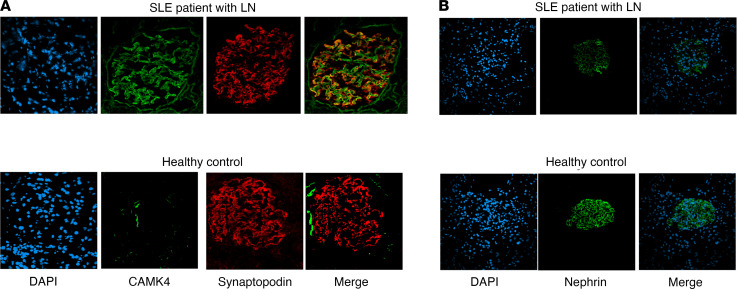
Individuals with active LN have increased CaMK4 expression in podocytes. (**A**) Representative images of immunofluorescence staining for synaptopodin and CaMK4 in glomeruli from kidney biopsies of patients with LN and controls without any glomerular lesion. Blue, DAPI; red, synaptopodin; Green, CaMK4. (**B**) Representative images of immunofluorescence staining for nephrin in glomeruli from kidney biopsies of patients with LN and controls without any glomerular lesion. Green, nephrin; blue, DAPI (*n* = 30 patients with LN and 5controls without any glomerular lesion).

Our in vitro data reveal a podocyte-specific pathogenic phenotype, which is elicited by aberrantly glycosylated IgG from patients with LN and is characterized by increased expression of CaMK4 and decreased expression of nephrin. To evaluate whether this phenotype is associated with glomerular disease in patients with SLE, we examined the expression of CaMK4 and nephrin in kidney biopsies from 30 patients referred to our center for suspected LN between 2017 and 2018. The baseline clinical characteristics of the studied patients are listed in Table 2. CaMK4 expression was increased in the glomeruli of patients with LN. Confocal immunofluorescence studies showed that CaMK4 colocalized with synaptopodin, a podocyte marker (69), demonstrating its presence in podocytes (Figure 7A). Consistent with our experimental data, the podocyte protein nephrin was decreased in CaMK4-overexpressing glomeruli from kidney biopsies of patients with LN compared with those of individuals who had undergone a kidney biopsy but had no identifiable glomerular lesion (control) (Figure 7B).

To determine whether the presence of CaMK4 in podocytes was associated with the histologic diagnosis of LN, we performed 2 different logistic regression analyses. We first adjusted for age, duration of disease, and use of immunosuppressive drugs, and we found that the presence of CaMK4 in podocytes predicted the presence of histologically proven LN (*P* < 0.001). Next, we adjusted for activity and chronicity indices, and we found that CaMK4 expression in podocytes was again significantly associated with the presence of LN (*P* = 0.005). CaMK4 expression was high in 27 of the 30 specimens of individuals who had been evaluated for suspicion of LN. Οne of the 3 CaMK4-negative specimens had a diagnosis of ANCA-associated vasculitis. The 2 other specimens had histologic evidence of LN Class V and Class VI without evidence of histologic activity. Upon further correlation analysis, CaMK4 expression was observed to be associated with histologic activity index (*r* = 0.5675). The correlation coefficients for the various factors considered are shown in Table 3, and the β (change per 1 SD) values for each factor are shown in Supplemental Table 1. These data reveal the value of detecting CaMK4 in kidney biopsy tissue as a surrogate marker for the presence of active LN.

### CaMK4 expression in urine podocytes identifies individuals with active LN.

Kidney biopsies are invasive with potential complications and are difficult to repeat to monitor disease activity during treatment. Therefore, we sought to determine whether the expression of CaMK4 in urine podocytes reflects active nephritis in patients with SLE. Urine from 15 individuals with SLE was collected. Four individuals did not have any kidney involvement, while 5 had a histologic diagnosis of active LN without any prior treatment and 6 had partial or complete clinical response to treatment after being initially diagnosed with LN. LN was diagnosed by kidney biopsy, as interpreted by a nephropathologist. Complete response was defined as improved proteinuria to < 500 mg/day, inactive urine sediment, and a serum creatinine within 20% of baseline. Partial clinical response was defined as a 50% reduction in proteinuria to less than 1.5 g/day and stable serum creatinine. Clinical characteristics of these individuals are displayed in Table 4.

**Figure 8:**
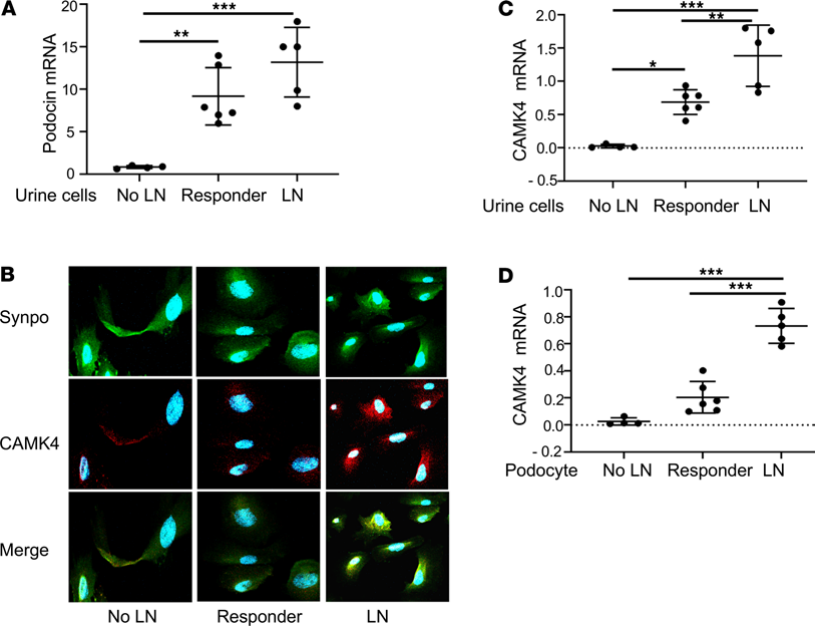
CaMK4 expression in urine podocytes identifies individuals with active LN. (**A**) Total urine podocin mRNA in individuals with SLE without nephritis (no LN), with active lupus nephritis (LN), and with LN who showed clinical response to treatment (responder). (**B**) Representative images of immunofluorescence staining for synaptopodin (green) and CaMK4 (red) in urine cells from an individual with SLE and no LN, an individual with LN and a responder. (**C** and **D**) Total urine CaMK4 mRNA (**C**) and urine podocyte CaMK4 mRNA (**D**) in individuals with SLE without nephritis (no LN) (*n* = 4), individuals with LN (*n* = 6), and responders. (*n* = 5).

The presence of podocytes in cytospun urine cells was detected by the presence of the podocyte markers podocin (by RT-PCR) and synaptopodin (immunofluorescence). Our data reveal that individuals with LN had a larger number of podocytes in the urine when compared with those without LN (Figure 8A). CaMK4 staining was positive in urine podocytes isolated from individuals with active LN (Figure 8B). Furthermore, total urine cell CaMK4 mRNA expression was elevated in individuals with active LN when compared with those with clinical response after treatment and those without kidney involvement (Figure 8C). Since the expression of CaMK4 in podocytes in kidney biopsy samples was associated with histologic diagnosis of LN, we sought to examine whether urine podocyte CaMK4 mRNA can differentiate between active and clinically inactive LN. Magnetic beads coated with antibodies to synaptopodin were used to isolate podocytes. CaMK4 mRNA levels were increased only in podocytes isolated from the urine of patients with active LN, while those with clinical response had minimal CaMK4 mRNA expression (Figure 8D). These findings, albeit in a small cohort of patients, support the diagnostic value of CaMK4 in isolated urine podocytes and introduce a potentially novel noninvasive approach to monitor LN disease activity.

## Discussion

In this communication, we demonstrate that CaMK4 is overexpressed in podocytes of individuals with active LN and that IgG from patients with SLE without kidney involvement does not have the ability to upregulate CaMK4, whereas IgG isolated from individuals with LN can injure podocytes through the upregulation of CaMK4. Furthermore, we found that CaMK4 is upregulated in urine podocytes from individuals with LN but not from healthy individuals or those with SLE without kidney involvement. Mechanistically, we demonstrate that CaMK4 represses nephrin transcription by phosphorylating NF-κB, which enhances the expression of the nephrin repressor SNAIL. Furthermore, we describe for the first time to our knowledge the importance of IgG N-glycans in the induction of CaMK4 and podocyte injury in LN. While the presence of core fucose renders IgG pathogenic, the presence of galactose has a protective effect, and its removal enhances the ability of IgG to injure podocytes (Supplemental Figure 7).

While the link between subclinical immune-mediated kidney injury and progressive kidney dysfunction is established, clinical evidence of LN appears later in disease presentation. Given the significant morbidity associated with LN, the ability to accurately identify individuals with SLE who are destined to develop kidney disease can significantly advance the field and avail a therapeutic window, which we presently lack. We rely on tissue biopsies to provide crucial information for treatment decisions, but biopsy material can be subject to sampling error, and their invasive nature limits repeated use. In this study, we demonstrate expression of CaMK4 in podocytes in tissue samples with active LN. We also show that CaMK4 mRNA levels in cells isolated from urine are elevated in individuals with LN when compared with individuals with SLE without evidence of kidney involvement. Interestingly urine podocyte CaMK4 expression levels were able to differentiate LN patients with ongoing active disease from those in remission. While these data need confirmation in a larger cohort of patients to establish urine cell expression of CaMK4 as a biomarker to detect LN, they are encouraging and propel us in a direction away from invasive diagnostic methods. More importantly, this may be helpful as a clinical tool to detect active flares and follow response to treatment, which remains challenging even today and relies heavily on clinical signs that have a significant lag period and do not correlate with histology. In parallel, we demonstrate a cell-based assay in which cultured podocytes upregulate CaMK4 only when exposed to IgG from patients with LN. This cell-based technique may also be useful in identifying feasible therapeutic targets for kidney disease in SLE that may be suitable for human clinical trials, in turn decreasing the need for animal-based molecule identification.

IgG produced in response to immunization undergoes N-glycan modification, which differs from normal serum IgG (70, 71). The ability to regulate levels of N-glycosylation likely relies on B cell intrinsic factors and would be subject to the immune milieu (71–75). The bulk of evidence comes from the fields of tumor immunology and the preparation of recombinant therapeutic antibodies, which — through proper glycoengineering — alter their properties. In addition, several studies have linked the degree of IgG-Fc glycosylation with the severity of antibody-mediated disease (76). The antiinflammatory activity of i.v. immunoglobulin treatment has been shown to be associated with the presence of sialic acid in a α2,6-linkage to a terminal galactose on IgG (72–74). Aberrant IgG glycosylation in patients with SLE has been observed with a possible proinflammatory role (77). Since podocytes are continuously exposed to IgG while clearing it from the basement membrane (78), it is possible that the N-glycans of the antibody may affect these cells in different ways. It was initially reported that Fc fragments, but not F(ab’)_2_ fragments, can bind to podocytes in culture and in tissue sections (79, 80). However, no specific receptors responsible for this binding were identified, and subsequent searches for the known Fcγ receptors CD16, CD32, and CD64 on podocytes yielded negative results (81–85). Podocytes have been found to express Fc receptor like protein 1 (FcRl1), which does not have a known ligand and does not bind IgG (86). Eventually, the presence of FcRn in podocytes was confirmed by flow cytometry, reverse transcription PCR (RT-PCR), and Western blotting. Furthermore, it was demonstrated that the binding of heat-aggregated IgG to podocyte lysates is pH dependent (85, 86). We have previously demonstrated that FcRn is essential for IgG-induced CaMK4 upregulation because it is needed for the entrance of IgG into the cell (29, 60). Prior studies have also shown that podocytes utilize FcRn for transcytosis to clear IgG from the glomerular basement membrane (78). IgG binds to FcRn as soon as the early endosome becomes acidic and permissive for pH-dependent interaction between FcRn and IgG. FcRn-bound IgG is subsequently sorted into common recycling endosomes, which recycle IgG away from lysosomes and back to the cell surface, where IgG is extruded into the extracellular milieu due to the neutral pH in that locale (87–97). Our data suggest that removal of glycans by PNGase F does not alter the ability of IgG to bind to FcRn or enter podocytes, which is consistent with prior studies showing that IgG glycosylation does not alter its capacity to bind to FcRn (95, 96). It is likely that both glycosylated and deglycosylated IgG after binding to FcRn traffic in the same way. However, it is certainly possible that differentially glycosylated IgG may lead to the formation of abnormal endosomes or lysosomes, which would be important to evaluate in future studies. In this study, we demonstrate that removal of N-glycans from IgG renders IgG incapable to induce CaMK4 expression in cultured podocytes. This property can be targeted by treatment with enzymes to remove these pathogenic glycan residues or by competitive inhibition of pathogenic IgG by glycoengineered IgG. We further found that SLE patients with nephritis have severely undergalactosylated IgG when compared with individuals with SLE without kidney disease or when compared with healthy individuals. It is plausible that this change occurs prior to the development of LN and, hence, may enlarge our window of identifying SLE patients on the cusp of developing LN.

Interestingly, we found that removal of fucose from IgG from patients with LN diminished podocyte injury induced by IgG. Galactose on the IgG exerted protection from injury in our experiments, and removal of galactose from IgG isolated from healthy individuals converted it into pathogenic IgG able to induce CaMK4, downregulate nephrin, and cause podocyte injury. Studies have also shown that circulating IgG from active patients with SLE have exposed fucose residues linked to disease activity (97). While information on fucose-induced signaling is limited, interaction of this moiety with lectins can enhance calcium flux in B cells (98). More recently dectin-1, a C-type lectin, has been identified as an endogenous ligand for core fucose on human IgG (99). Future studies defining the pattern of glycosylation should be of great interest, as it may enable the development of novel therapeutics to decrease the need for global immunosuppression. Genetic loci associated with N-glycosylation of human IgG have also been identified in autoimmune diseases in GWAS (100). Interestingly, the identified loci (*ST6GAL1*, *B4GALT1*, and *FUT8*) encode glycosyltransferases associated with fucose and galactose addition or transferring sialic acid residues to galactose residues (100). These data, along with the evidence we present here, suggest that understanding the effect of differential N-glycosylation on immunoglobulin function can contribute to the design of more effective therapies involving treatment of patients with glycan modifiers.

Podocytes share many elements of the innate and adaptive immune system and contribute to inflammation in LN (21). Specifically, they produce and express complement components and receptors, which — when dysregulated — appear to contribute to podocyte damage and LN (101–106). In parallel, podocytes express major histocompatibility complex and costimulatory molecules, which may be involved in local immune events (107–109). We have shown that antibodies present in lupus sera enter podocytes to upregulate CaMK4, which in turn compromises their structure and function, and that treatment targeting this molecule only in the podocyte prevents LN in mice (19, 29). CaMK4 is responsible for regulation of CD86 in podocytes, along with other cytokines that are key players in autoimmunity (29). Podocyte injury appears to occur early in LN, while proteinuria and progressive glomerulosclerosis often persist despite increased immunosuppression (71). An approach that would involve targeted delivery of a CaMK4 inhibitor to podocytes (19) or aim to ameliorate loss of podocytes could be proposed to treat patients with LN. This would not only obviate the need for large doses of drugs systemically, but also limit the use of drugs that cause global immunosuppression.

In summary, we demonstrate that IgG isolated from patients with LN, in contrast to the IgG isolated from SLE patients without LN, can lead to podocyte injury. We found that LN IgG elicits podocyte-specific pathogenic signaling that involves upregulation of CaMK4, phosphorylation of NF-κB, upregulation of SNAIL, and repression of nephrin transcription. The pathogenic properties of LN IgG depend on the presence of N-glycans because their removal deprives it of its capacity to cause podocyte injury. Specifically, fucose removal from LN IgG makes it nonpathogenic, while galactose removal renders IgG from healthy controls pathogenic. The mechanism of podocyte injury described here offers a potentially novel insight on the role of IgG N-glycans in podocyte signaling in LN and suggests potentially novel therapeutic considerations. Further, albeit in a small cohort of patients, we introduce 2 “liquid biopsy” approaches to detect and follow disease activity; in the first, cultured podocytes can be exposed to serum IgG and determine whether it causes increased expression of CaMK4, and in the second, urine podocytes can be tested for the expression of CaMK4. Both approaches may avert the need for kidney biopsy for the diagnosis and following response to treatment.

## Methods

### Patients and controls.

We studied 30 patients (18–65 years old) who were referred to our center for kidney biopsy between 2017 and 2019 to evaluate the presence of LN. Deidentified clinical and pathologic information was extracted from biopsy reports for patients whose biopsies were included. Five tissue samples were obtained from individuals who underwent a kidney biopsy but had no identifiable glomerular lesion. We also analyzed the urine and serum of 15 additional patients who fulfilled at least 4 of the 11 American College of Rheumatology revised criteria for the classification of SLE (110–112). All patients were women between the ages of 20 and 64 years and had SLE disease activity index scores ranging from 8 to 16 (113). Serum samples were collected and stored at −80°C until used. Fresh urine was collected and evaluated as specified below.

### IgG purification.

IgG purification kits (Dojindo Molecular Technologies) were used to purify IgG from SLE patients or healthy volunteers according to the manufacturer’s protocol. Purity was confirmed by SDS-PAGE.

### Immortalized human podocyte cell line.

The immortalized human podocyte cell line was cultured as previously described (29). Briefly, cells were cultured with RPMI-1640 with 10% FBS, insulin, transferrin, and selenium. These cells proliferate at 33°C and differentiate into mature podocytes in 7–10 days after transfer to 37°C due to the temperature-sensitive SV40-T gene and a telomerase gene. Ten days after being transferred to 37°C, cells were treated with IgG (10 μg/mL) from patients with SLE with active LN, patients with SLE without kidney involvement, or healthy controls. These cells were collected 24–72 hours after stimulation and analyzed by Western blotting.

### Western blotting.

Podocytes were lysed in RIPA buffer or NP40 lysis buffer at 4°C for 30 minutes. After centrifugation (16,400*g*; 30 minutes; 4°C), supernatants were collected and an identical amount of protein from each lysate was separated on NuPAGE 4%–12% Bis-Tris Gel (Thermo Fisher Scientific). Proteins were transferred to a nitrocellulose membrane, which was subsequently blocked for 1 hour using 5% nonfat dry milk or 3% BSA in TBS-T and incubated at 4°C overnight with mouse anti–human CaMK4 (catalog 610276/clone 26, BD Biosciences), anti-NF-κB (p65) (D14E12, Cell Signaling Technology [CST]), anti-nephrin (GP-N2, Progen), mouse anti–human SNAIL (L70G2, CST), and mouse anti–human GAPDH (catalog 649202/FF26A/F9, BD Biosciences). The membrane was washed with TBS-T and incubated with anti–rabbit or anti–mouse IgG coupled with HRP (catalogs sc-2004, sc-2020, sc-2005, sc-2473, respectively; Santa Cruz Biotechnology Inc.). The ECL system (Amersham) was used for detection. Bands on blots corresponding to proteins of interest were analyzed by ImageJ software (NIH).

### Real-time PCR.

Total mRNA was isolated from human podocytes, or sorted urine podocytes using the RNeasy Mini Kit (QIAGEN), and then cDNA was synthesized using cDNA EcoDry Premix (Clontech) for PCR amplification. Real-time PCR analysis was performed with the Light Cycler 480 System (Roche) using TaqMan gene expression assays according to the manufacturer’s specifications (Applied Biosystems). Expression was normalized to GAPDH. All primers and probes were from Applied Biosystems and were as follows: *CaMK4* (Mm01135329_m1), *NPHS1* (Hs00190446_m1), and *GAPDH* (Hs02786624_g1). Gene expression was assessed by the comparative Ct method.

### Transfecting with small interfering RNA (siRNA).

Human podocytes were transfected with *CaMK4* siRNA (Thermo Fisher Scientific), *FCRN* siRNA (Thermo Fisher Scientific), or NF-κB (p65) (CST) or control siRNA (Thermo Fisher Scientific) using INTERFERin transfection reagent (Polyplus Transfection) according to the manufacturer’s protocol. After 24 or 48 hours of incubation, the cells were exposed to IgG and then collected for RNA extraction or were stained for immunofluorescence analysis.

### Immunofluorescence.

Frozen kidney sections (4 μm) were fixed in 4% formaldehyde for 10 minutes or acetone for 3 minutes and blocked for 1 hour in BSA, followed by overnight incubation with mouse anti–human CaMK4 antibody (catalog 610276/clone 26, BD Biosciences) or goat anti–human synaptopodin antibody (catalog sc25137/p-19). Next, sections were washed and stained for 1 hour with Alexa Fluor 488– or 568–labeled donkey anti-goat or anti-mouse antibodies (catalog A21432/ A21206, Invitrogen). Finally, DAPI or Hoechst 33258 (Invitrogen) was applied for nuclear staining.

Cultured podocytes (0.2 × 10^5^) were seeded onto type I collagen 4-well culture slides (BD Biosciences) and exposed for 24 hours to fluorescence-labeled IgG or nonlabeled IgG (10 μg/mL) from LN patients and healthy individuals. After 30 minutes of incubation with RPMI 1640 medium supplemented with 2% BSA, the cells were washed once with PBS and fixed for 20 minutes with 4% paraformaldehyde. The cells were permeabilized for 5 minutes with 0.1% Triton X-100 (Thermo Fisher Scientific) in PBS, followed by blocking for 30 minutes with PBS containing 2% BSA. Cells were then stained for 1 hour at room temperature with anti-FcRn antibody (1:100 dilution; Santa Cruz Biotechnology Inc.). After washing 3 times with PBS, the cells were stained with Alexa Fluor 568 anti–rabbit IgG (highly cross-absorbed) as the secondary antibody (catalog A21202, Invitrogen). The stained cover glasses were mounted on a glass slide with 10 μL of DAPI Fluoromount-G (Southern Biotech) and sealed with nail polish.

Stained specimens were analyzed with a Nikon Eclipse Ti confocal microscope. Images were analyzed with EZ-C1 v.3.7 software, and fluorescence intensities or areas were measured by ImageJ software.

### Cell isolation from urine.

Urine was spun at 1500 rpm for 5 minutes, and the supernatant was discarded. PBS was used to resuspend the sediment and was then exposed to antibody-coated magnetic beads with rotation for 10 minutes at room temperature. The antibodies that were used were directed against 3 podocyte-specific proteins: nephrin, podocin, and synaptopodin. A magnet was used to separate the cells (podocytes) from the beads. Cells were isolated from beads.

### Deglycosylation of IgG.

PNGase F, α-fucosidase, neuraminidases S and A, and β-galactosidase were purchased from New England Biolabs, and deglycosylation was performed following the manufacturer’s protocol without denaturing the protein.

### Mass spectrometry analysis of IgG N-glycans.

Approximately 7 μg of purified IgG of each sample were loaded into an SDS-page gel (4%–12%). After staining with Coomassie Brilliant Blue, the bands corresponding to the IgG heavy chain (~50 kDa) were excised. The gel pieces were washed with a solution of 50% acetonitrile in 50 mM ammonium bicarbonate (AMBIC), briefly dried with a vacuum centrifuge (70*g*, 10 minutes, room temperature), and incubated with 200 μL of 10 mM 1,4-Dithiothreitol (DTT) for 30 minutes at 50°C. The DTT solution was then discarded, and the gel pieces were washed with acetonitrile and briefly dried. In total, 200 μL of 55 mM iodoacetamide (IAA) was added to the samples and incubated 30 minutes at room temperature in the dark. The IAA solution was next discarded, and the samples were washed with 50 mM AMBIC, followed by acetonitrile, before briefly drying the gel pieces. The samples were then incubated with 500 μL of a TPCK-treated trypsin solution (20 μg/mL in 50mM AMBIC) at 37°C overnight. The supernatants were recovered in new tubes before carrying out 2 sequential washes with 200 μL of 50 mM AMBIC, vortexed for 15 minutes; 200 μL of 50% acetonitrile in 50 mM AMBIC, vortexed for 15 minutes; and 200 μL of acetonitrile, vortexed for 15 minutes. For each sample, all washes were collected, pooled in the same tube, and lyophilized.

The dried materials were resuspended in 200 μL of 50 mM AMBIC, to which 1 μL of PNGase F was added for an overnight incubation at 37°C. The released N-glycans were purified over a C18 Sep-Pak (50 mg) column (Waters) conditioned beforehand with 1 column volume (CV) of methanol, 1 CV of 5% acetic acid, 1 CV of 1-propanol, and 1 CV of 5% acetic acid. The C18 column was washed with 5% acetic acid, flow through; wash fractions were collected, pooled, and lyophilized.

Lyophilized N-glycan samples were incubated with 1 mL of a DMSO–NaOH slurry solution and 500 μL of methyl iodide for 30 minutes under vigorous shaking at room temperature. A total of 1 mL of Milli-Q water (MilliporeSigma) was then added to stop the reaction, followed by 1 mL of chloroform to purify the permethylated N-glycans. The chloroform fractions were washed 3 times with 3 mL of Milli-Q water. The chloroform fractions were dried before being redissolved in 200 mL of 50% methanol and were then loaded into a conditioned C18 Sep-Pak (50 mg) column with 1 CV of methanol, 1 CV of Milli-Q water, 1 CV of acetonitrile, and 1 CV of Milli-Q water. The C18 columns was washed with 3 mL of 15% acetonitrile and then eluted with 3 mL of 50% acetonitrile. The eluted fractions were lyophilized and then redissolved in 10 μL of 75% methanol, from which 1 μL was mixed with 1 μL 2,5-dihydroxybenzoic acid (DHB) (5 mg/mL in 50% acetonitrile with 0.1% trifluoroacetic) and spotted on a MALDI polished steel target plate (Bruker Daltonics).

MS data were acquired on a Bruker UltraFlex II MALDI-TOF Mass Spectrometer instrument. The reflective positive mode was used, and data were recorded between 500 and 6000 *m/z*. For each MS N-glycan profile, the aggregation of 20,000 laser shots or more were considered for data extraction. Only MS signals matching an N-glycan composition were considered for further analysis. Subsequent MS postdata acquisition analysis was made using mMass (112). The relative abundance of each N-glycan identified was calculated based on the absolute intensity of the first isotopic peak of a given N-glycan relative to the sum of all N-glycan intensities.

### AAL ELISA.

Ninety-six–well microtiter plates were coated with 2 mg/mL anti–human IgG in coating buffer overnight. Plates were then blocked with deglycosylated blocking buffer (deBSA; BSA treated with sodium periodate to oxidize any sugars present). After blocking, serum IgG was diluted at 1:1000 in TBS-Ca-Mg and 0.05% Tween-20 (Thermo Fisher Scientific) and incubated at 37°C for 2 hours. These serum dilutions resulted in a saturating binding of IgG to the ELISA plate. After every incubation step, the plates were washed 3 times with 200 mL TBS-Ca-Mg-Tween. To detect exposed glycosyl residues on IgG, biotin-labeled AAL (50 ng/mL, Vector Laboratories) was added at room temperature for 1 hour. After washing, the plates were incubated with HRP-streptavidin (The Jackson Laboratory) at the recommended concentration for 1 hour at room temperature. Detection was performed for both types of ELISA with the addition of substrate solution. Optical density values were obtained by ELISA-plate reader employing a 450 nm/620 nm filter/reference pair. This method was adapted from prior studies (113).

### Transwell migration assay.

Transwell cell culture inserts (pore size, 5 μm; Costar Corp., Corning) were coated with type I collagen, rinsed once with PBS, and placed in multiwell plates filled with RPMI medium. For each experiment, 2 × 10^4^ podocytes were seeded in the inserts and allowed to migrate for 24–72 hours at 37°C. Nonmigratory cells were removed from the upper surface of the membrane, and migrated cells were fixed with cold methanol and stained with Crystal Violet Solution (MilliporeSigma). The migrated cells were counted with a 20× objective in the center of a membrane (1 field).

### Phalloidin staining.

Podocytes were seeded onto type I collagen 4-well culture slides (BD Biosciences) and cultured for 24–72 hours. The cells were then washed once with PBS and fixed for 20 minutes with 4% paraformaldehyde. They were permeabilized for 5 minutes with 0.1% Triton X-100 in PBS, followed by blocking for 30 minutes with PBS containing 2% BSA; they were stained for 30 minutes at room temperature with AF488-labeled phalloidin (A12379, Thermo Fisher Scientific). After washing 3 times with PBS, Hoechst 33258 staining was done; then, the slide was sealed with nail polish. Stained specimens were analyzed with a Nikon Eclipse Ti confocal microscope. Images were analyzed with EZ-C1 v.3.7 software, and fluorescence intensities of actin fibers were measured by ImageJ software.

### Statistics.

Statistical analyses were performed with GraphPad Prism version 7.0 software and STATA version 15. Statistical significance was determined by *t* test (2-tailed) for 2 groups, 1-way ANOVA with Bonferroni multiple comparisons tests, or 2-way ANOVA with Bonferroni’s multiple comparisons tests for 3 or more groups. *P* < 0.05 was considered statistically significant. For kidney biopsy data, the descriptive characteristic of the examined population of patients was prepared, determining minimum, maximum mean, and median values. The study variables were analyzed using the logistic regression model. The model facilitates the examination of the impacts of multiple independent variables on a binary dependent variable Y. The values of variable Y were coded as follows: 1, presence of a particular trait; and 0, absence of a particular trait. Correlation between the presence of LN and CaMK4 in podocytes was determined by Pearson correlation test.

### Study approval.

Human kidney biopsies were collected at the Beth Israel Deaconess Medical Center. The protocol concerning the use of biopsy, serum, and urine samples from patients with LN was approved by the IRBs on human subjects at Beth Israel Deaconess Medical Center (no. 088-2015).

## Author contributions

RB and GCT conceived and planned the experiments. RB, KM, and SL carried out the experiments. IES, SK, and LE contributed to sample preparation and collection. RDC, RB, GCT, MGT, and MP contributed to the interpretation of the results. RB, MGT, and GCT wrote the manuscript. All authors provided critical feedback and helped shape the research, analysis, and manuscript.

## Supplementary Material

Supplemental data

## Figures and Tables

**Table 1:**
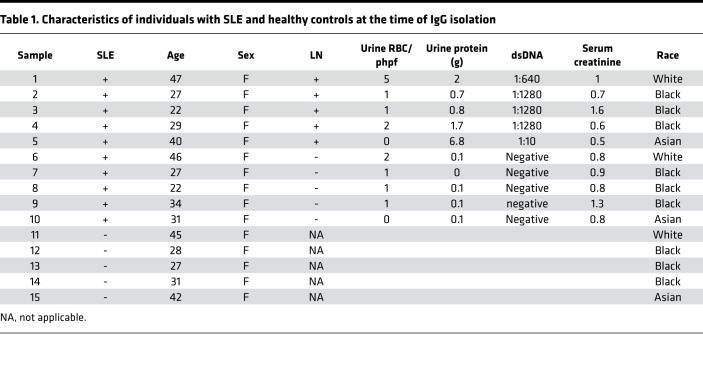
Characteristics of individuals with SLE and healthy controls at the time of IgG isolation

**Table 2:**
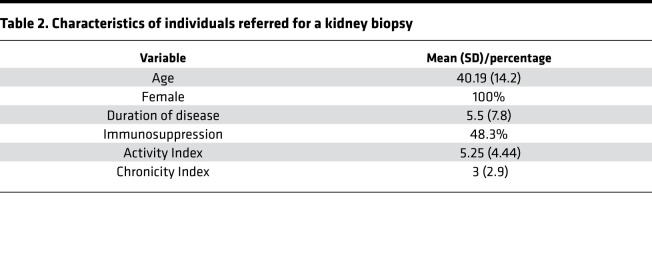
Characteristics of individuals referred for a kidney biopsy

**Table 3:**
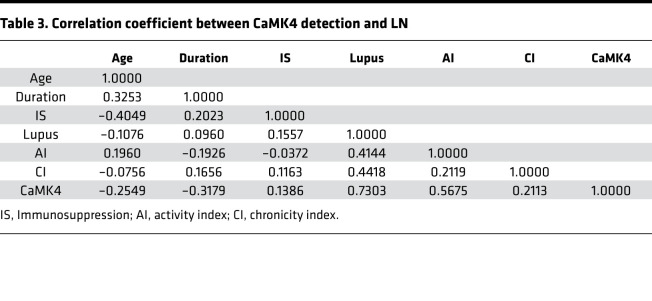
Correlation coefficient between CaMK4 detection and LN

**Table 4:**
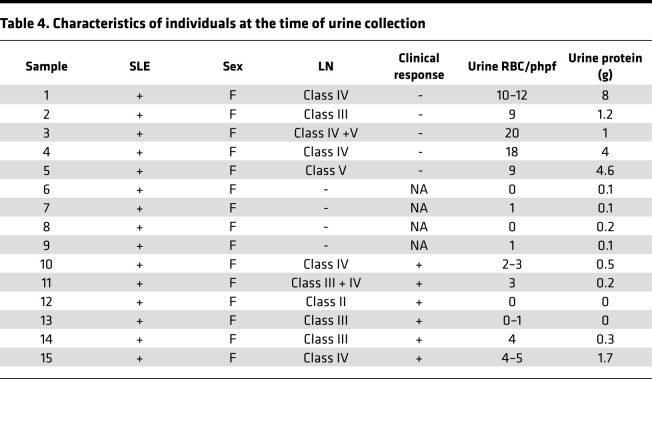
Characteristics of individuals at the time of urine collection

## Version 1 03/30/2021

In-Press Preview

## Version 2 05/10/2021

Electronic publication
