# Supplementary material for: Aberrantly glycosylated IgG elicits pathogenic signaling in podocytes and signifies lupus nephritis
Source: JCI Insight. 2021 May 10;6(9):e147789. doi: 10.1172/jci.insight.147789 (PMC8262331; doi:10.1172/jci.insight.147789)
Supplement: Supplemental data [file jciinsight-6-147789-s293.pdf]

Supplementary Data:

Supplementary Table I: β coefficients of variables used in multivariate regression analysis.

Variable	β
CAMK4	.4955
Age	.0048
Duration	-.0067
Immunosuppression	.00176
Activity Index	-0.026
Chronicity Index	-.0015
Cons	.7553

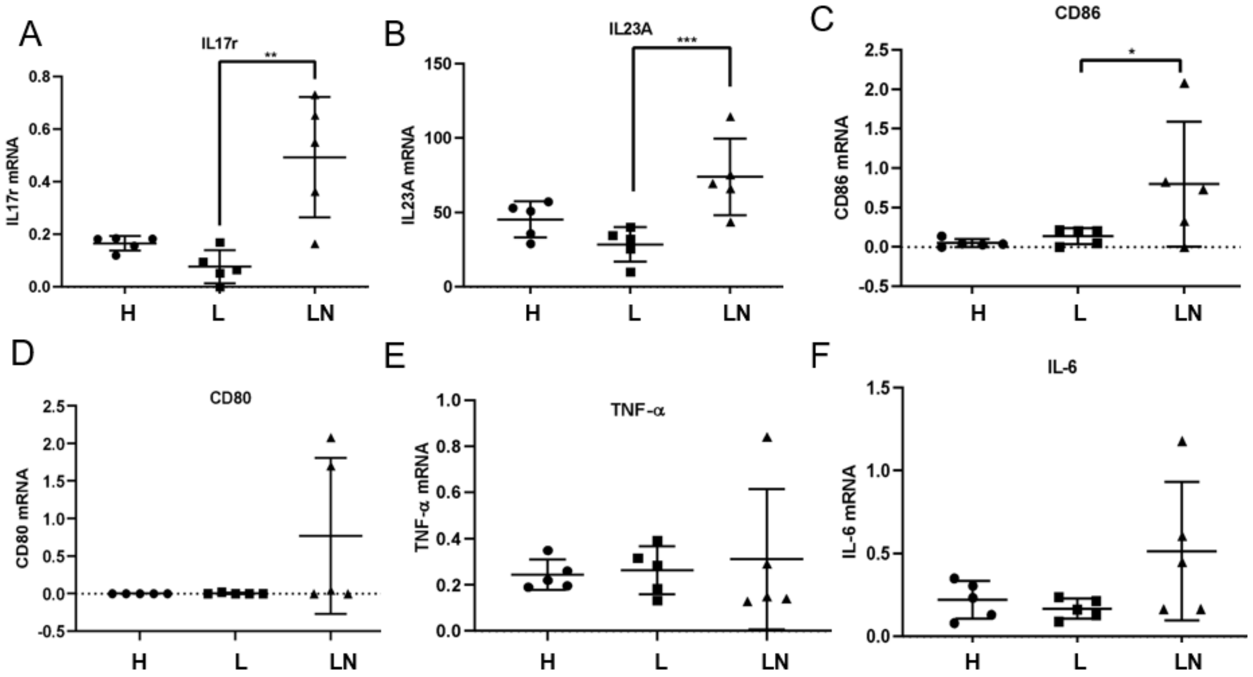

Supplementary Fig. 1: IgG from patients with lupus nephritis (LN) causes upregulation of different genes in podocytes compared to those with SLE without kidney disease (L) and healthy controls(H).

(A) IL-17r, (B) IL23A, (C) CD86, (D) CD80, (E) TNF-alpha, (F) IL-6 mRNA expression in podocytes after exposure to IgG from SLE patients with no kidney involvement (L), active LN(LN) and healthy controls(H). (n=15)

A

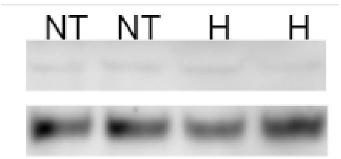

B

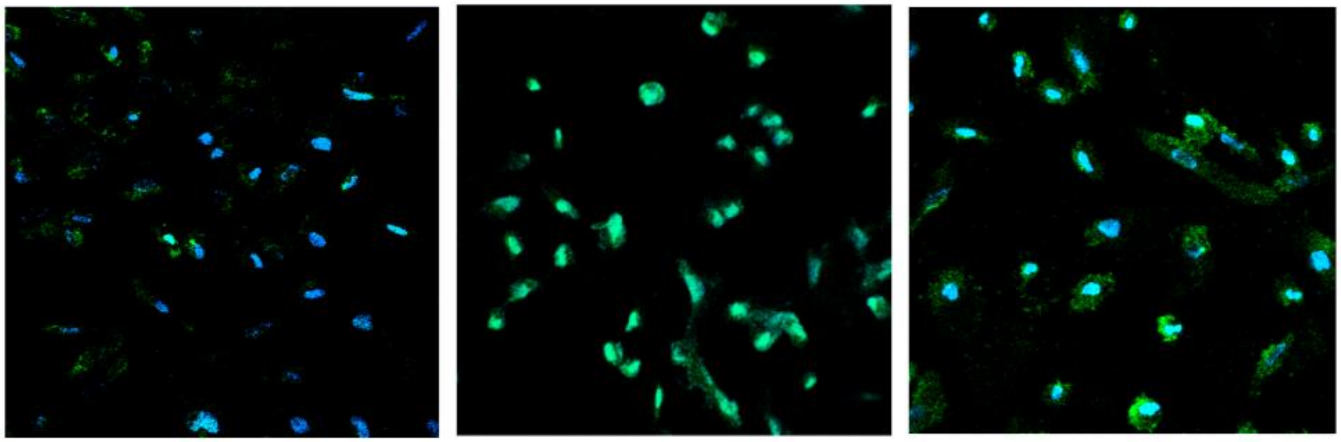

Supplementary Figure 2 : (A) CaMK4 is not upregulated in cultured podocytes (NT) or after exposure to IgG derived from healthy volunteers (H). (B) CaMK4 is detected mostly in the nucleus 30 minutes after exposure to LN IgG and in both the cytoplasm and the nucleus after 24 hours of exposure to LN IgG.

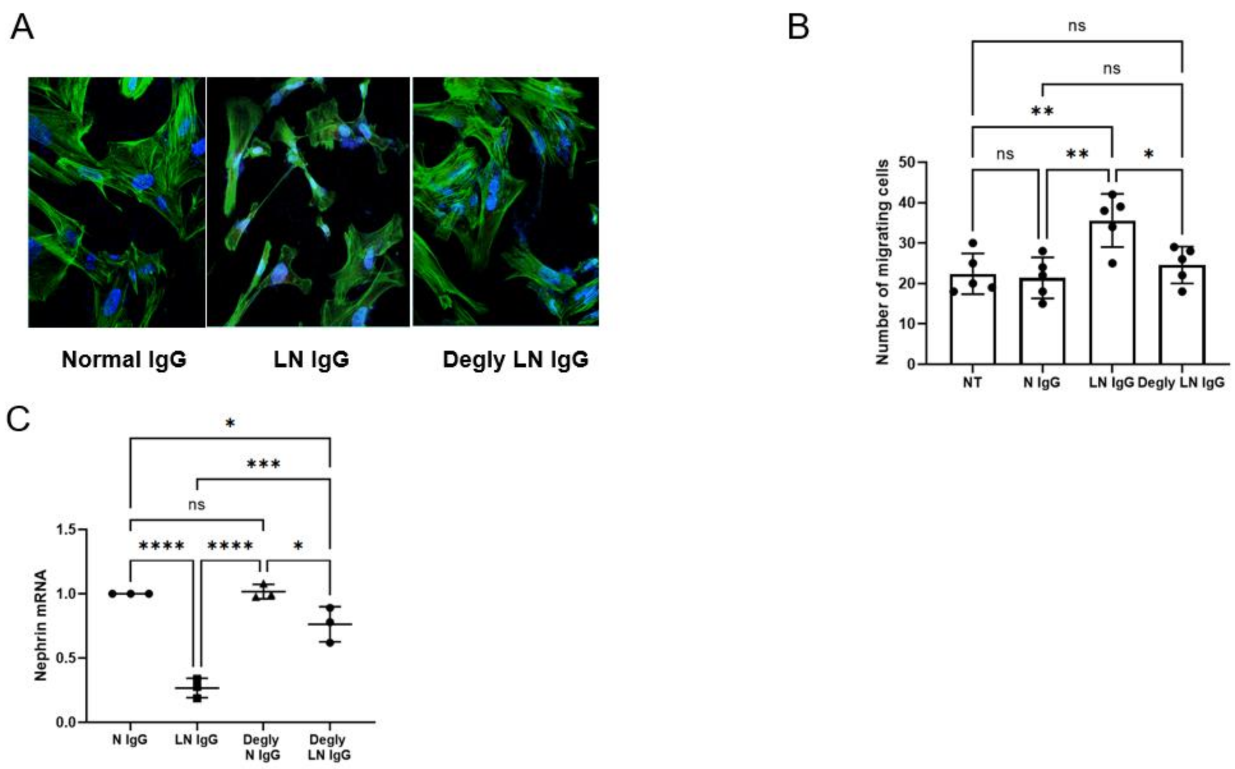

Supplementary Figure 3. Deglycosylation of LN IgG prevents actin cytoskeleton and motility changes in podocytes (A) Representative results of phalloidin staining of human podocytes after exposure to IgG from controls or patients with LN with or without treatment with PNGase (Deglycosylated IgG= Degly IgG). (B) Result of Transwell migration experiments using human podocytes treated with control or LN IgG with or without treatment with PNGase. Error bars represent mean ± SEM (n = 5 independent experiments). *P < 0.05, One way ANOVA test. (C) Removal of N-glycans from LN IgG prevents nephrin repression in podocytes

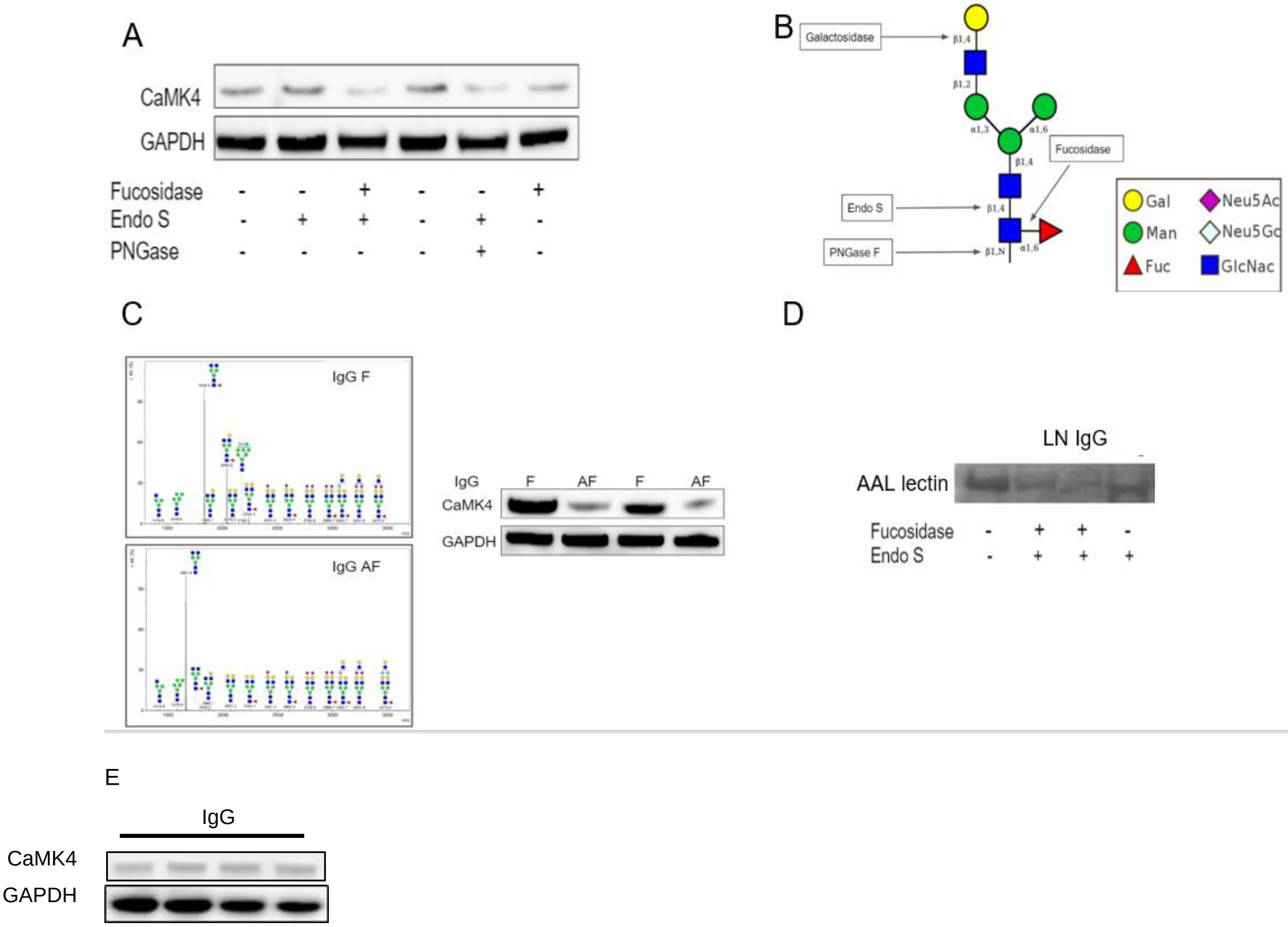

Supplementary Figure 4: IgG induced CaMK4 upregulation depends on fucose attached to the Fc segment of the antibody.

A: LN IgG was treated with fucosidase, Endo S and PNGase prior to podocyte exposure. CaMK4 expression was evaluated by western blot.

B: A schematic representing the site of action of each enzyme used.

C: CaMK4 expression was measured in podocytes after exposure to fucosylated (F) and afucosylated (AF) IgG. The N-linked glycan analysis for these IgG is displayed above.

D: AAL Lectin Blot after treatment of LN IgG with and without fucosidase and Endo S.

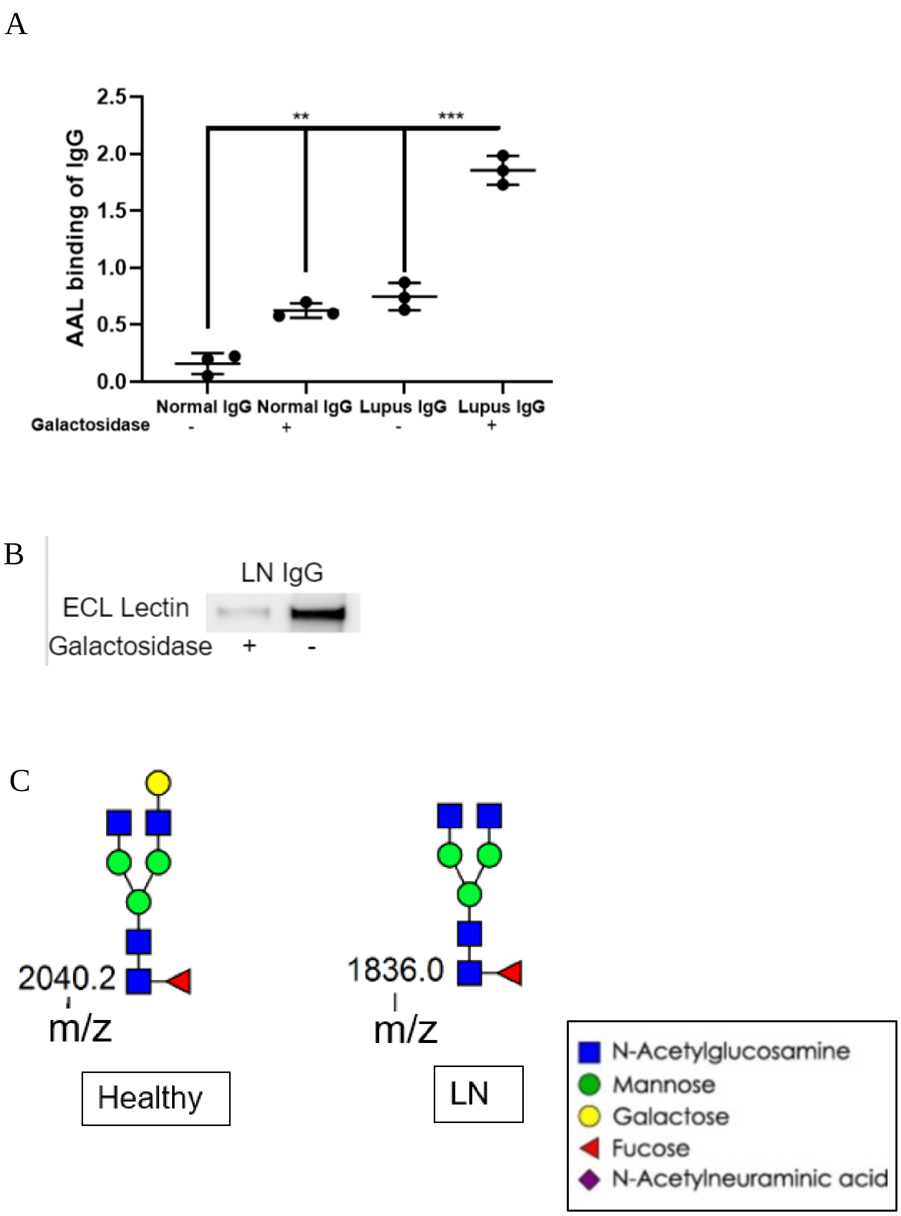

Supplementary Figure 5: AAL binding to fucose on IgG is decreased in the presence of galactose in a lectin ELISA assay. A) An ELISA was performed with AAL lectin (specific for fucose) to evaluate binding of IgG bound fucose from healthy individuals and individuals with LN pre- and post-treatment with galactosidase. B) Galactose removal was confirmed by ECL (Erythrina Cristagalli) blot. C) The dominant glycoform on IgG in LN and controls is depicted. IgG in all groups share a common core structure including the first two N-acetylglucosamine residues , then the three mannose residues and eventually the terminal N-acetylglucosamine residues. These glycoforms also have a core fucose attached to the first N-acetylglucosamine residue. A terminal galactose is noted in IgG from healthy controls while most LN IgG lacks this terminal galactose. These two glycoforms are almost equally prevalent in IgG from SLE without nephritis and LN in remission.

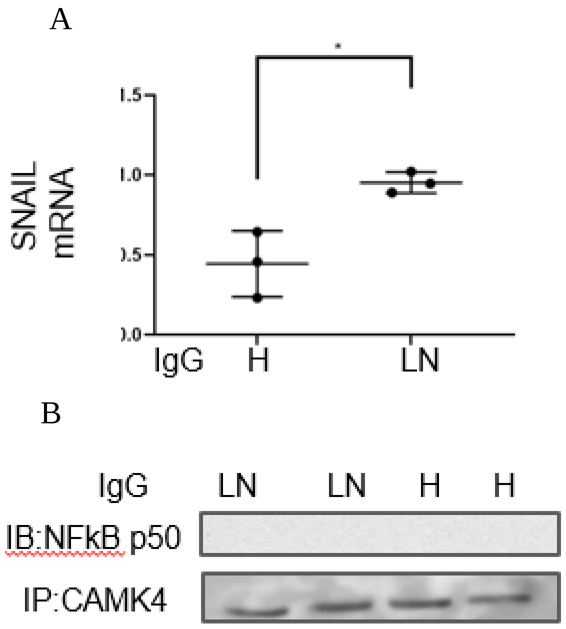

Supplementary Figure 6. A: SNAIL mRNA is increased in podocytes after exposure to IgG from individuals with LN compared to healthy controls(H). B: NFκB p50 does not interact with CaMK4. CaMK4 was immunoprecipitated from podocytes after exposure to IgG from individuals with LN and healthy controls and immunoblotted (IB) with an antibody against p50.

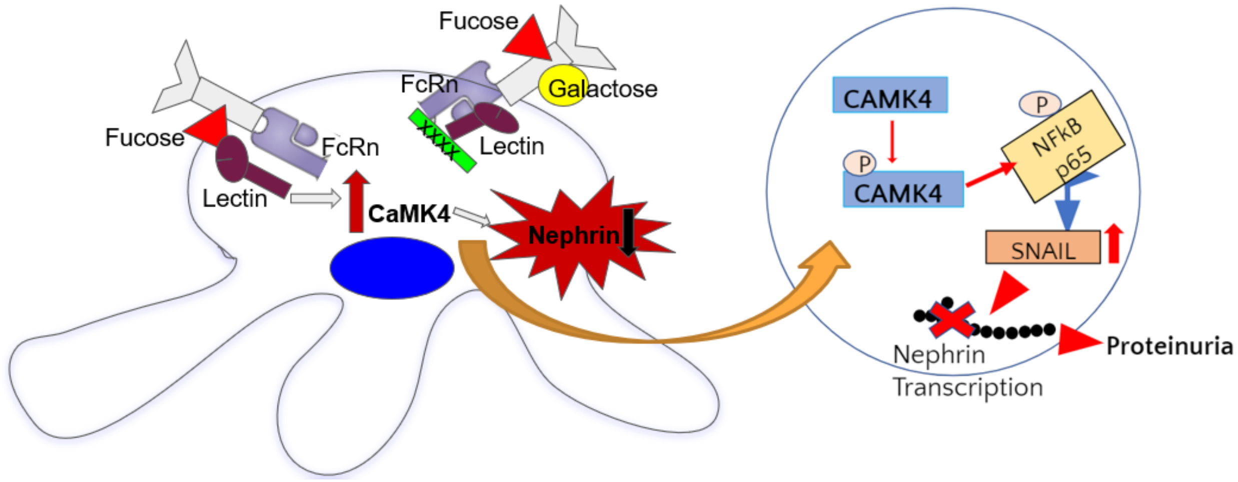

Supplementary Figure 7: Schematic representation of LN IgG-mediated podocyte injury. Under-galactosylated IgG in LN increases CaMK4 in podocytes which in turn suppresses nephrin transcription.
